# Novel protocol to observe the intestinal tuft cell using transmission electron microscopy

**DOI:** 10.1242/bio.059007

**Published:** 2022-02-16

**Authors:** Takuma Kozono, Miwa Tamura-Nakano, Yuki I. Kawamura, Takashi Tonozuka, Atsushi Nishikawa

**Affiliations:** 1 Institute of Global Innovation Research, Tokyo University of Agriculture and Technology, Tokyo 183-8509, Japan; 2 Communal Laboratory, Research Institute, National Center for Global Health and Medicine, Tokyo 162-8655, Japan; 3 Department of Gastroenterology, The Research Center for Hepatitis and Immunology, Research Institute, National Center for Global Health and Medicine, Chiba 272-8516, Japan; 4 Department of Applied Biological Chemistry, Graduate School of Agriculture, Tokyo University of Agriculture and Technology, Tokyo 183-8509, Japan

**Keywords:** Tuft cell, Transmission electron microscopy, Correlative light-electron microscopy, Aclar film

## Abstract

The tuft cell is a chemosensory cell, a specific cell type sharing the taste transduction system with a taste cell on the tongue, of which the existence has been discovered in various tissues including the gastrointestinal tract, gall bladder, trachea and pancreatic duct. To date, electron microscopic approaches have shown various morphological features of the tuft cell, such as long and thick microvilli, tubulovesicular network at the apical side and prominent skeleton structures. Recently, it has been reported that the small intestinal tuft cell functions to initiate type-2 immunity in response to helminth infection. However, the mechanisms by which such distinguished structures are involved with the physiological functions are poorly understood. To address this question, a combination of physiological study of tuft cells using genetic models and its morphological study using electron microscopy will be required. However, it is a challenge to observe tuft cells by electron microscopy due to their extremely low frequency in the epithelium. Therefore, in this paper, we suggest an advanced protocol to observe the small intestinal tuft cell efficiently by transmission electron microscopy using serial semi-thin sections on Aclar film.

This article has an associated First Person interview with the first author of the paper.

## Abstract (precis)

**Summary:** We suggest an advanced protocol to efficiently observe the small intestinal tuft cell, a rare cell on the intestinal epithelium, by transmission electron microscopy.

## INTRODUCTION

The tuft cell is a chemosensory cell that exists as a small population at the mucosal tissues with morphological features such as longer and thicker microvilli at their apical surface. The tuft cell was first reported in rat trachea in 1956 (Rhodin and Dalhamn, 1956). Since then, it has been discovered that tuft cells are distributed throughout the body, in the glandular stomach (Jarvi and Keyrilainen, 1956), gastric groove (Wattel and Geuze, 1978), small intestine (Isomäki, 1962; 1973), cecum (Okamoto et al., 2008), colon (Silva, 1966), gall bladder (Luciano and Reale, 1990), bile duct (Luciano et al., 1981), pancreatic duct (Kugler et al., 1994; Bailey et al., 2014; Delgiorno et al., 2014; Schütz et al., 2019), submandibular gland (Sato and Miyoshi, 1997), auditory tube (Krasteva et al., 2012), thymic medulla (Panneck et al., 2014) and urethra (Deckmann et al., 2014; Deckmann and Kummer, 2016). In addition to mammals including primates, carnivora, perissodactyla, artiodactyla and rodentia (Deckmann et al., 2015), tuft cells are also conserved in fish (Podkowa and Goniakowska-Witalińska, 2002) and frogs (Sugimoto et al., 1983). Intriguingly, immunohistochemical analyses have shown that the tuft cells distributed in various tissues express similar genes (Yamashita et al., 2017). In particular, they share taste transduction-related genes with taste cells on the tongue, hence they are also called taste chemosensory cells (Yamashita et al., 2017). Furthermore, recent reports based on RNA-sequence analysis of the sorted tuft cell and single-cell RNA-sequence analysis also verified the high similarity in the gene expression profile among thymic, tracheal, and gall bladder, small intestinal and colonic tuft cells, although there is a subtle heterogeneity (Haber et al., 2017; Bornstein et al., 2018; Miller et al., 2018; Nadjsombati et al., 2018). With these, we can imagine the potential importance of tuft cells in the sensory system to detect the environmental signals in various organisms.

**Fig. 1.**
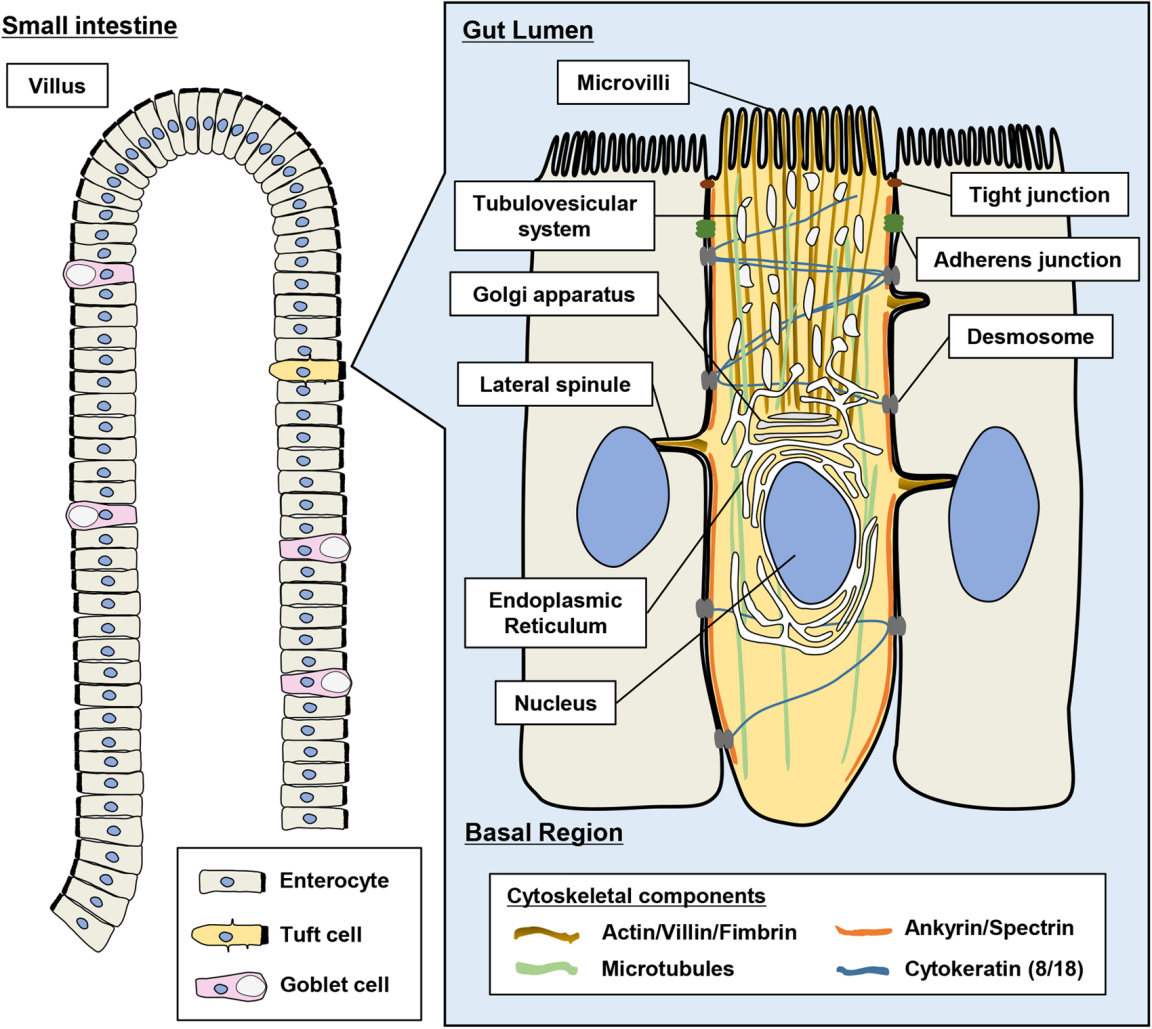
**Schematic representation showing the low frequency of tuft cells on the intestinal epithelium and structure of the murine tuft cell.** Long and thick microvilli exist at the tip of the tuft cell. The lateral spinules are attached to the nucleus of the neighboring cells. The filaments (actin/villin/fimbrin) extend from the tip of the microvilli to the periphery of the nucleus. Microtubules are longitudinally arranged from the tip of the microvilli to the basal region. Intermediate filaments consisting of cytokeratin 8/18 and cell junctions: tight junctions, adherens junctions and desmosomes anchor the tuft cell to the neighboring cells. Ankyrin/spectrin structure undercoats along the cell membrane.

To date, electron microscopic and immunohistochemical approaches have provided knowledge of the morphological features of tuft cells: long and thick microvilli on the apical side, a tubulovesicular network that extends from the microvilli to the periphery of the Golgi apparatus, lateral spinules that protrude to the neighboring cells and prominent skeletal structures, as shown in Fig. 1 (Wattel and Geuze, 1978; Höfer and Drenckhahn, 1996; Höfer et al., 1999; Saqui-Salces et al., 2011; Hoover et al., 2017). Hoover et al., 2017 reported using serial block-face (SBF) and automated tape-collecting ultra-microtome (ATUM) scanning electron microscopy (SEM), a new electron microscopic system which allows us to observe the target cell by volume rendering of serial sections, that the tubulovesicular network probably connects with the endoplasmic reticulum. They also showed that three to four lateral spinules in each tuft cell are projected into the cytoplasm and nucleus of the neighboring cell. They speculated that the functions of the tubulovesicular network and lateral spinules might be exchanges of the molecular cargos with the intestinal lumen and neighboring cells, respectively. In addition to knowledge accumulation regarding the morphology and gene expression patterns of the tuft cell, three groups recently reported that the small intestinal tuft cell has a role in detecting parasitic infection and inducing type-2 immunity to expulse the worms (Gerbe et al., 2016; Howitt, et al., 2016; von Moltke et al., 2016). According to the new reports including theirs, the small intestinal tuft cells secrete the interleukin-25 (IL25) and leukotriene to the lamina propria to induce type-2 immunity on detecting the infection (Luo et al., 2019; McGinty et al., 2020). However, we have not achieved a complete understanding of the molecular mechanism by which the tuft cells detect the infection and transduce the signal. Particularly, an outstanding question is how the morphological characters including distinguished microvilli, prominent skeletal structures, and arrangements and structures of organelles are involved with physiological function. For example, we speculate that the long and thick microvilli are important for catching the targets such as molecules and parasites efficiently. Furthermore, the prominent skeletal structures might support the distinguished structures of microvilli or transport system of the unknown secreted vesicles in the tuft cells. For further understanding, it will contribute to linking physiological study using genetic models and morphological study using electron microscopy. However, it is still very difficult to prepare the ultrathin sections containing tuft cells due to their extremely low frequency in the epithelium.

As previously reported, correlative light-electron microscopy (CLEM) is a strong tool which makes it possible to find semi-thin sections containing a small population of target cells such as tuft cells followed by the preparation for the ultrathin sections (Luciano and Reale, 1990). In their report, the tuft cells on the epithelium of the gall bladder are distinguished by staining of the semi-thin sections with Toluidine Blue and the ultrathin sections are prepared from continuous face of the same block. Here, we tried to prepare the serial ultrathin sections from the serial semi-thin sections for the three-dimensional (3D) ultrastructural analysis of whole cells by re-embedding all the serial semi-thin sections containing the tuft cells on glass slides. However, there is a difficulty in transferring semi-thin sections on glass slides to the tip of the block by re-embedding. For example, when the semi-thin sections are re-embedded in epoxy resin and detached from the glass slides by broiling or acute cooling with liquid nitrogen (Pignot-Paintrand and Bressac, 1992; Sawaguchi et al., 2009), sometimes the semi-thin sections are partially left on the glass slides and are broken. These failures often damage the tissue structures and prevent efficient preparation for observation by electron microscopy. In particular, when serial ultrathin sections are prepared from the serial semi-thin sections containing target cells such as tuft cells, it is a serious problem because all the sections need to be re-embedded to keep the continuity of the ultrathin sections. In this paper, we introduce an advanced CLEM method, which has no concerns about damage to the samples on re-embedding by preparing the serial semi-thin sections on the Aclar film, hence, our method successfully avoids the loss of the semi-thin sections containing tuft cells. Our new protocol also enables us to prepare the serial ultrathin sections of the tuft cell from the serial semi-thin sections, which will be useful for investigating the intracellular position and shape of each organelle, the organelle–organelle interactions, and the interactions between organelle and plasma membrane by transmission electron microscopy (TEM), without using high-end equipment such as SBF-SEM or ATUM-SEM. Our advanced method will make it more efficient to observe tuft cells by TEM, which will promote studies on the relationship between the morphological characters and physiological functions of tuft cells at molecular levels.

## RESULTS AND DISCUSSION

### A novel CLEM strategy for preparing serial ultrathin sections of intestinal tuft cells using Aclar film

**Fig. 2.**
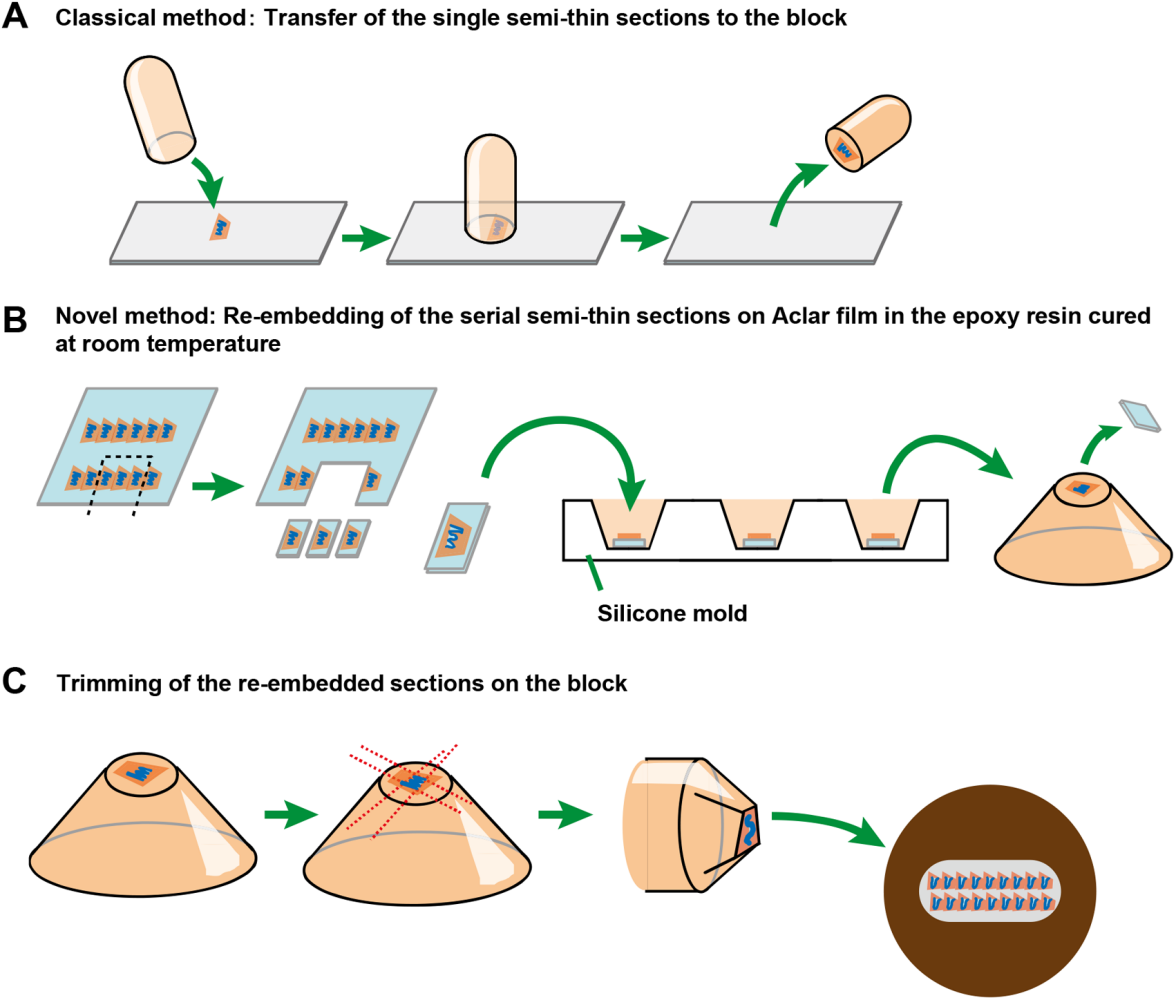
**Schematic representation showing the CLEM strategy.** (A) Classical protocol to re-embed a single semi-thin section on glass slides after identification of the position of the targets by light microscopy. (B) Re-embedding the serial semi-thin sections without space on the Aclar films for the preparation of serial ultrathin sections after identification of the position of the target by light microscopy. Each semi-thin section was cut out and re-embedded in silicone mold. (C) Re-embedded section can be trimmed to pick up many ultrathin sections on one grid.

The small intestinal tuft cell constitutes less than 1.0% of the epithelium at a steady state, which makes it very difficult to identify the position of the tuft cell on observing ultrathin sections by TEM. As a basic classical CLEM method, the semi-thin sections (1–2 µm) are placed on glass slides and the position of the target to observe is then identified by light microscopy. Only semi-thin sections containing the target are then transferred from the glass slides to the tip of the blocks by re-embedding (Fig. 2A) (Sawaguchi et al., 2009). However, we need to take great care on transferring the semi-thin section on glass slides to avoid tissue damage, as we mentioned above. Particularly, tuft cell can be seen in three to four serial semi-thin sections, probably due to its size and distorted morphology, which means that it is necessary to succeed in the transfer of all of the serial semi-thin sections containing the tuft cell for 3D ultrastructural analysis of whole cell. Here, we tried to solve this difficulty by preparing the semi-thin sections on Aclar film, a clear film resistant to heat and embedding reagents, which can be easily removed from the polymerized blocks without the failure after re-embedding described above, unlike glass slides. Furthermore, the serial semi-thin sections can be lined up in several rows with no space on the Aclar film and the Aclar film on which the semi-thin sections are placed is easily cut out due to its physical properties (Fig. 2B). Thereby, each of the serial semi-thin sections can be re-embedded one by one. The re-embedded sections can be then trimmed into small area containing the identified tuft cell, which allows as many serial ultrathin sections as possible to be picked up on one grid (Fig. 2C). Based on this concept, we validate this advanced method to observe the small intestinal tuft cell efficiently by TEM.

### The cost-effective and noninvasive identification process of the position of intestinal tuft cells by Toluidine Blue staining

**Fig. 3.**
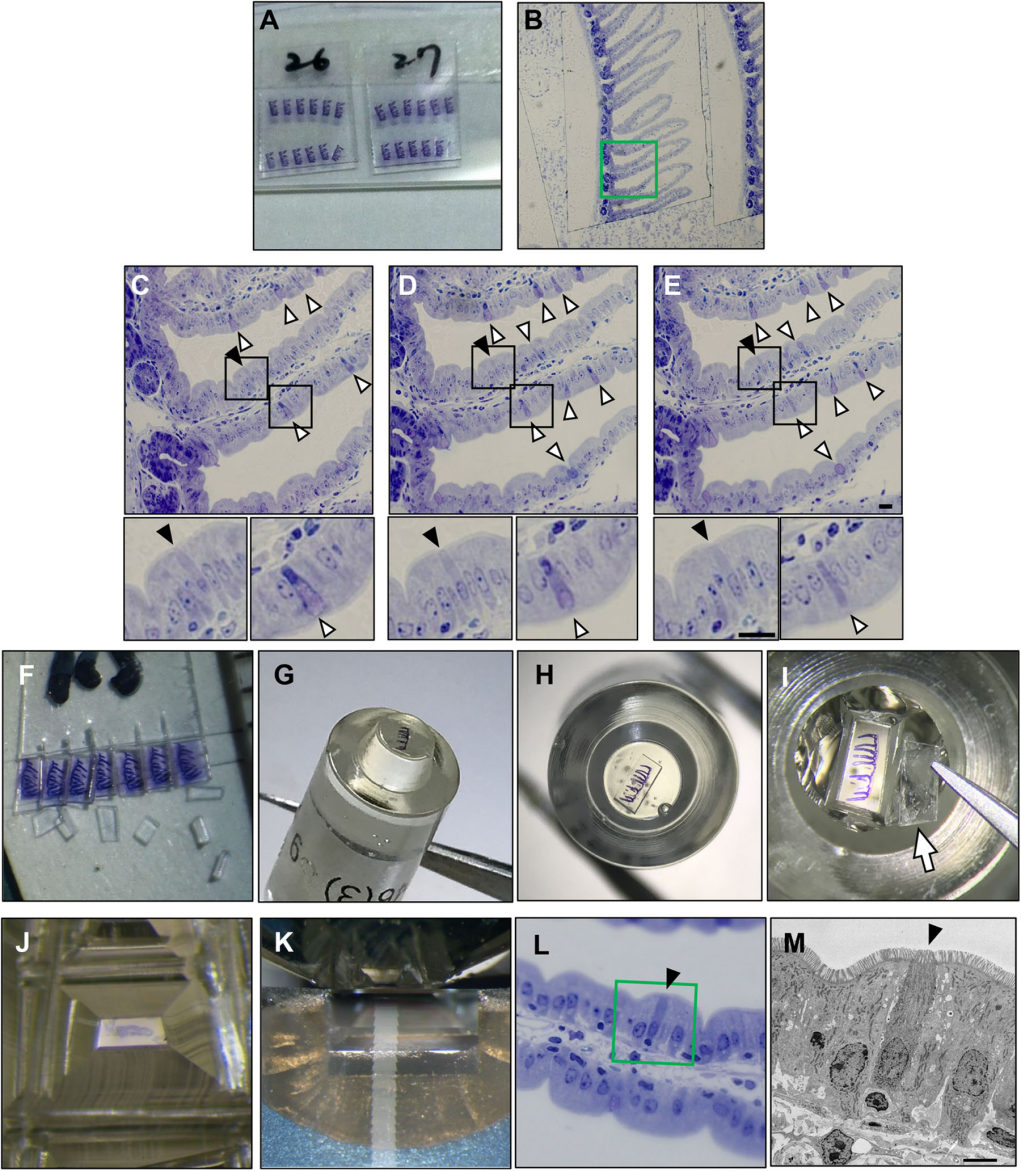
**Identification of the tuft cells on the semi-thin sections and re-embedding to prepare serial ultrathin sections.** (A) Ribbons of serial semi-thin sections attached to Aclar films. (B–F) Histological images of the small intestinal semi-thin sections stained with Toluidine Blue. A semi-thin section (B) and serial semi-thin sections corresponding to the frame colored in green in the image of B (C–E). Closed triangles, tuft cell; opened triangles, goblet cell. Scale bar: 10 µm. The magnified images correspond to the frame colored in black in each image of C–E. (F) Serial semi-thin sections on the Aclar film were cut into individuals. (G–K) A re-embedded resin block (G,H) was trimmed to a small area (J) after the Aclar film was removed (I). After that, the block was serially ultrasectioned (K). An Aclar film removed from the resin block is indicated by the arrow in I. (L) Histological images of the small intestinal semi-thin sections stained with Toluidine Blue. (M) A TEM image of the ultrathin section prepared from the semi-thin section in (L). The image corresponds to the frame colored in green in the image in L. Scale bar: 5.0 µm. (L,M) Closed triangles show the tuft cell.

To date, several studies describing the morphological features of the tuft cells using electron microscopy have adopted the CLEM strategy to identify the position of the tuft cells on the epithelium (Hoover et al., 2017; Kuga et al., 2017). Although the reporter mice were used to label the tuft cells in the work by Hoover et al., the establishment and use of gene-edited models to identify the position of tuft cell are time consuming and costly. On the other hand, Kuga et al. used a tuft-cell-specific antibody. However, labeling with antibodies requires antigen retrieval treatment, which often disrupts the tissue structure. Thus, it is also crucial to establish the low-cost and noninvasive CLEM strategy to observe the small intestinal tuft cells. Intriguingly, it has been reported that Toluidine Blue specifically stains the tuft cell in the gall bladder (Luciano and Reale, 1990). In accordance with the CLEM strategy, the semi-thin sections of the small intestine were stained with Toluidine Blue and the positions of tuft cells were confirmed by light microscopy before re-embedding (Fig. 3A,B). As shown in the microscopic images of serial semi-thin sections, the tuft cells were stained more densely with Toluidine Blue than the surrounding epithelial cells and were recognized as flask-shaped, microvillus-lined cells at the brush border of the epithelium (Fig. 3C–E, closed triangles). Toluidine Blue also deeply stained the goblet cells as shown in the previous reports (Fig. 3C–E, opened triangles) (Heazlewood et al., 2008; Yamada et al., 2002), but they were easily distinguished from the tuft cells by the characteristic unstained apical area of the large cell body containing numerous secretory granules. After this identification process, only the semi-thin sections containing tuft cells were then re-embedded and used for the preparation of the serial ultrathin section. Thus, although our protocol using Toluidine Blue is based on a classical TEM method, it would be a cost-effective and noninvasive procedure to identify the positions of the tuft cells because it does not require labeling the targets with the reporter genes or antibodies.

### Re-embedding semi-thin sections and trimming into small areas for efficient ultrastructural analysis of whole tuft cells

To prepare the ultrathin sections, the semi-thin sections on the Aclar film were re-embedded into the resin followed by transferring to the tip of the block as shown in Fig. 2B. Although we first tried to re-embed the semi-thin sections on the Aclar film in Quetol812, an epoxy resin that can be cured by thermal polymerization, the sections were peeled off the Aclar film during curing and were floated into the resin. For the preparation of the ultra-thin sections from the semi-thin sections, it is necessary that the semi-thin sections are kept horizontal on the bottom of the block. Therefore, we re-embedded the sections in NER-814, which is an epoxy resin cured at room temperature but not by thermal polymerization. Thereby, the semi-thin sections were re-embedded on the bottom of the block planarly (Fig. 3G,H). In no cases were the semi-thin sections on the Aclar film folded during re-embedding.

**Fig. 4.**
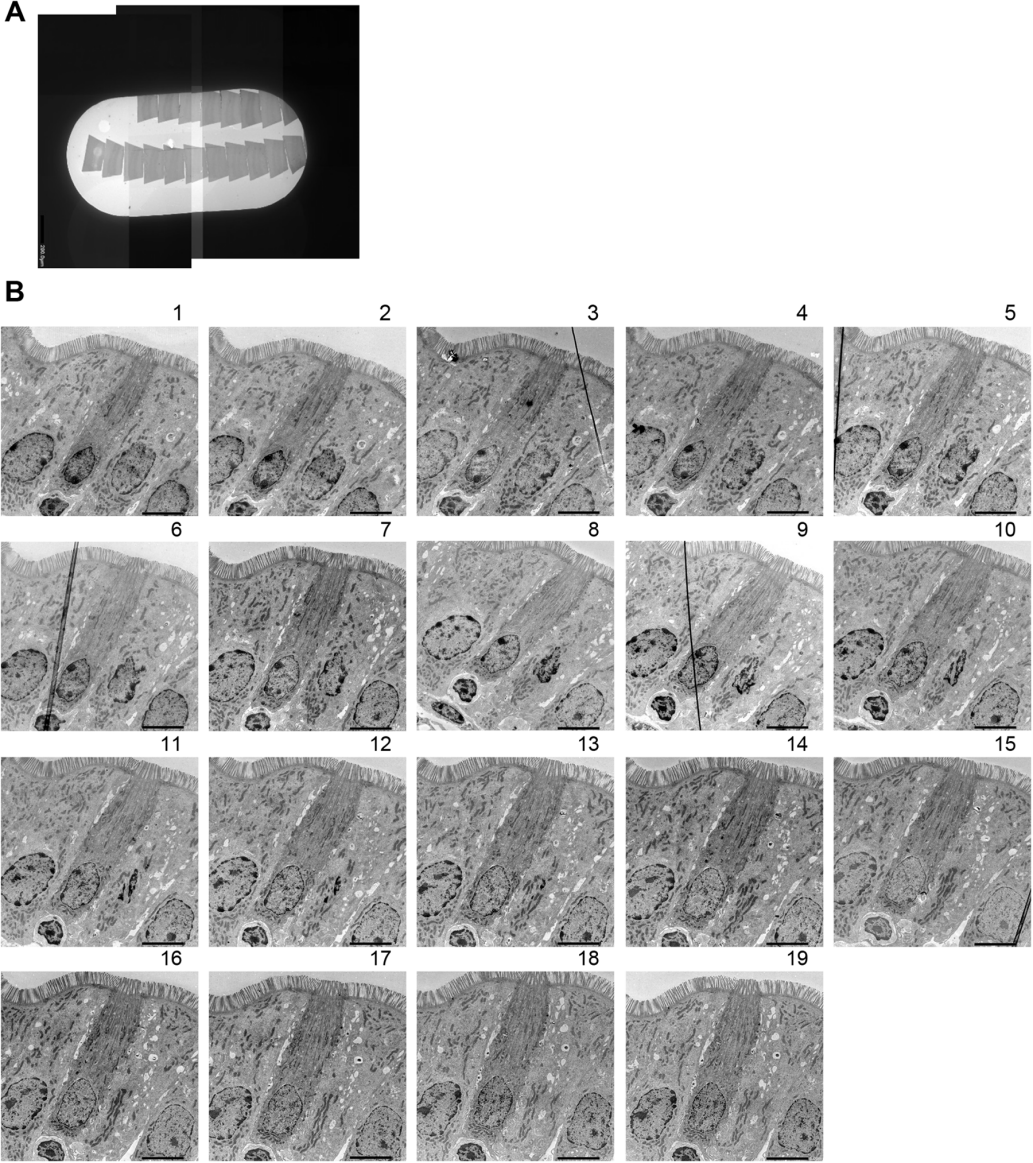
**Observation of the tuft cells using serial ultrathin sections by TEM.** (A) The serial ultrathin sections on a grid prepared from the semi-thin section. Images created by photomontage. (B) Images of the tuft cell in each section of the serial ultrathin sections in A. Scale bar: 5.0 µm.

The identification of the region containing tuft cells on light microscopy enabled us to minimize the semi-thin sections by trimming them into small areas (approximate ∼200 μm×150 μm) containing approximately one villus with identified tuft cells (Fig. 3J), as shown in Fig. 2C. The position of the villus was confirmed by visual search under microscopy, referring to the image of the semi-thin section captured by light microscopy before re-embedding. This trimming process allowed as many serial ultrathin sections as possible to be picked up on one grid (Fig. 4A). Here, the serial ultrathin sections were picked up on the grid so that each ultrathin section contained the region of interest with the tuft cell on which the position was identified on the semi-thin section. As shown in Fig. 4B, one tuft cell on the serial ultrathin sections, which was detected on semi-thin section stained with Toluidine Blue, was serially observed by TEM. Thus, this trimming process enabled efficient observation of the serial ultrathin sections by TEM with a smaller number of the grids.

### Observation of the tuft cell by TEM

**Fig. 5.**
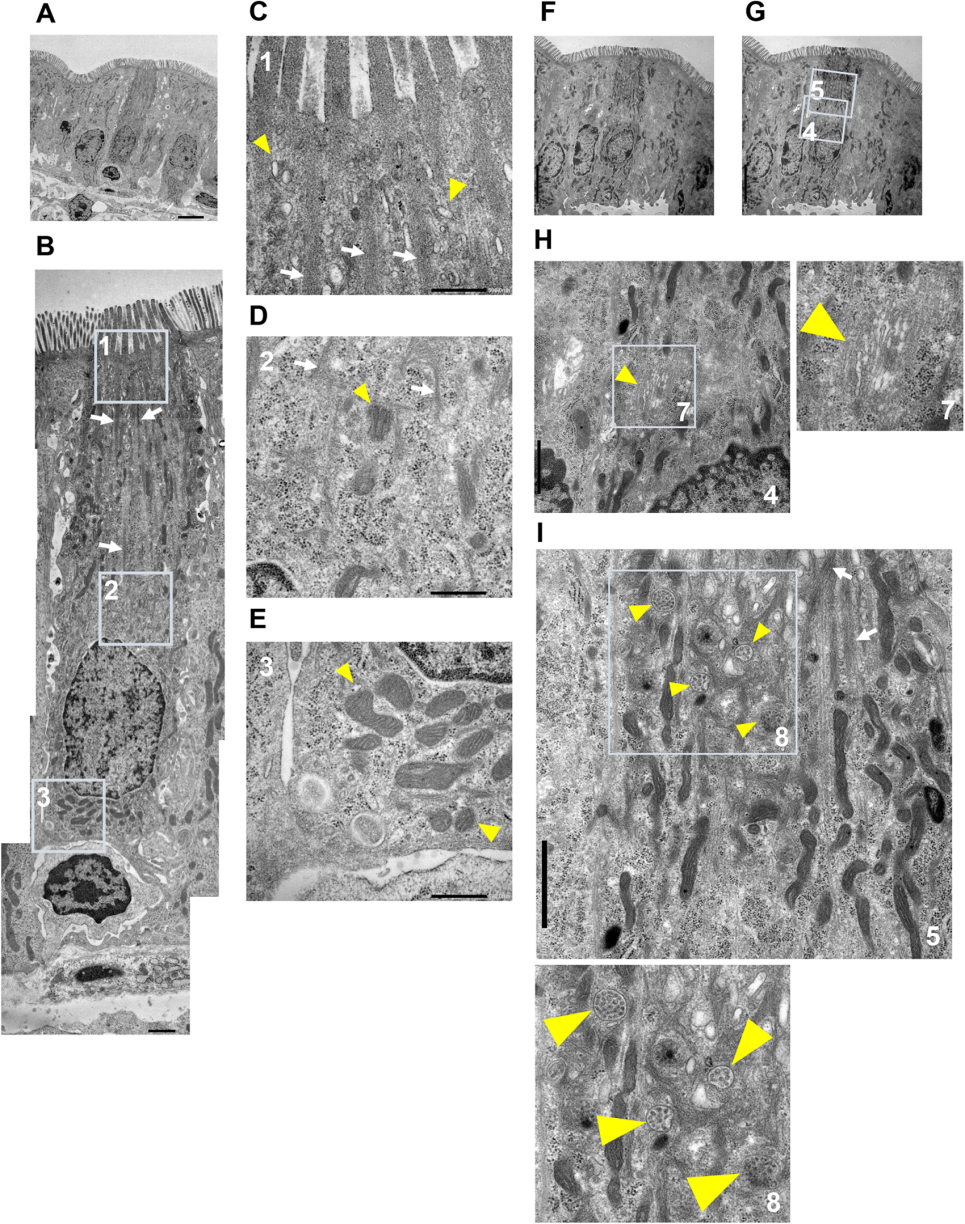
**Observation of the intracellular structure in the tuft cell by TEM.** (A) A TEM image of the tuft cell. (B) Images created by photomontage. Each area surrounded with box are shown as magnified images in C–E. (C–E) The yellow arrowheads show tubulovesicular system (C), centrosome (D) and basal mitochondria (E). (F,G) A TEM image of the tuft cell. Each area surrounded with box in the G are shown as magnified images in H and I. (H,I) The yellow arrowheads show Golgi apparatus (H) and multivesicular bodies (I). Each area surrounded with box in the image of (H,I) are shown as magnified images in the right (H) or below (I). The white arrows in (B–D,I) show the filamentous structures. Scale bars: 5.0 µm (A,F,G); 1.0 µm (B,H,I); 500 nm (C,D,E).

In this mouse, 19 out of 21 cells identified on the semi-thin sections by Toluidine Blue staining were tuft cells, exhibiting specific morphological features such as thick microvilli, when we observed by TEM. Although whole cell bodies in four out of these 19 cells were not possible to observe, it is probably due to their distorted morphology. Furthermore, these two cells without thick microvilli out of 21 cells were also tuft cells because other features of the tuft cells such as tubulovesicular system and distinguished filamentous structures were confirmed. Thus, all of the 21 cells identified on the semi-thin sections by Toluidine Blue staining were tuft cells on the observation by TEM. In particular, the position of the cell identified on the semi-thin sections by Toluidine Blue staining corresponded to that of the tuft cell observed in the electron micrograph and vice versa (Fig. 3L,M). Thus, the prior identification process on the semi-thin sections using Toluidine Blue certainly made it more efficient to find the tuft cells on the ultrathin sections on TEM. Furthermore, high-magnification observation showed the intracellular structures specific to tuft cells such as the microvilli (Fig. 5A–C,F,G) and tubulovesicular system (Fig. 5C), consistent with previous reports (Wattel and Geuze, 1978; Hoover et al., 2017). Particularly, filamentous structures extending from the tip of the cell to the periphery of the nucleus are distinguished (Fig. 5B–D,I, white arrows), consistent with the previous reports describing the high expression of specific cytoskeletal markers in tuft cells (Höfer and Drenckhahn, 1996; Saqui-Salces et al., 2011). Other organelles including the centrosome (Fig. 5D), basal mitochondria (Fig. 5E) and Golgi apparatus (Fig. 5H) were also confirmed. Intriguingly, the multivesicular bodies (MVBs) on the apical side of the tuft cells were also observed (Fig. 5I). Although these MVBs were concentratedly located on the apical side, their physiological significances are unknown.

### Observation of the tuft cell stimulated by succinate

**Fig. 6.**
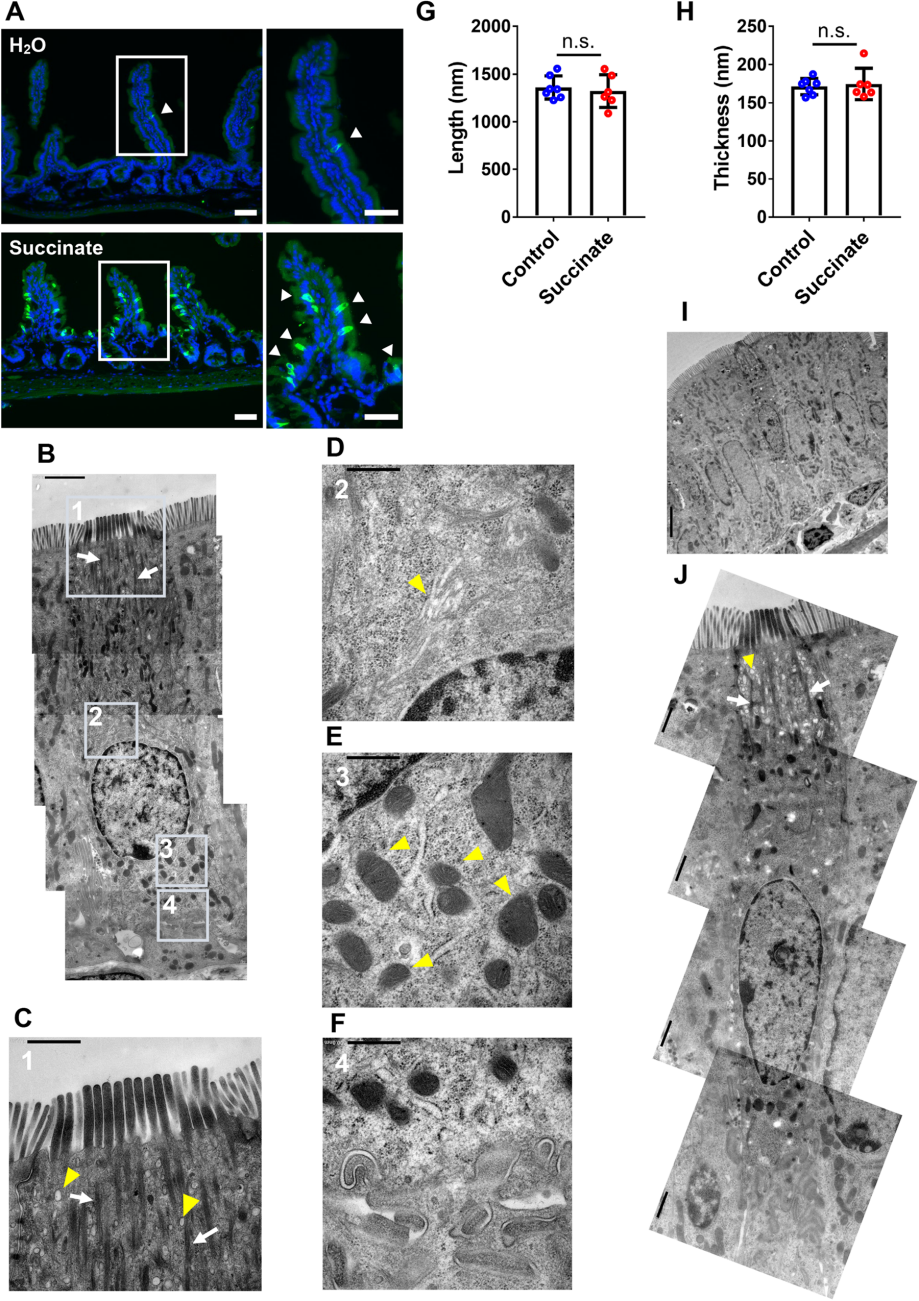
**Observation of the intracellular structure in the tuft cell stimulated by succinate.** (A) Immunofluorescence image of small intestine in the mice administrated with H_2_O or 200 mM succinate for 7 days. An anti-DCLK1 rabbit antibody (green) was used as a primary antibody. Nuclei were stained with Hoechst33342 (blue). The arrowheads show the tuft cells. Each area surrounded by a white box is shown as a magnified image on the right. (B) Images of the tuft cell in the mice administered with 200 mM succinate for 7 days created by photomontage. Each area surrounded with the boxes is shown as a magnified image in C–F. (C–E) Yellow arrowheads indicate the tubulovesicular system (C), Golgi apparatus (D) and basal mitochondria (E). (F) Basal region of the tuft cells in B. (G,H) The graphs showing the length (G) and thickness (H) of microvilli in each tuft cell. Each dot shows the average calculated from more than three microvilli in each tuft cell (control, *N*=7 tuft cells; succinate, *N*=6). Error bars show the s.d.; n.s., not significant; two-tailed Student’s *t*-test. (I) TEM image of the tuft cell from mice administered with 200 mM succinate for 7 days. The tissues were not stained with Toluidine Blue. (J) The images of the tuft cell in I were created by photomontage. Yellow arrowheads indicate the tubulovesicular system. The white arrows in B, C and J indicate the filamentous structures. Scale bars: 50 µm (A); 5.0 µm (I); 2.0 µm (B); 1.0 µm (C,J); 500 nm (D,E,F).

It has been reported that the intestinal tuft cells specifically express succinate receptor 1 (Sucnr1), and succinate stimulates the tuft cell followed by secretion of the IL25, resulting in the activation of type-2 immunity and expansion of the tuft cell (Lei et al., 2018). However, how the morphological changes in the activated tuft cells compare with those in the steady state was not known. Therefore, the mice were administered with succinate for 7 days and the intracellular structures of the stimulated tuft cells were investigated. Immunohistochemical analysis using an anti-DCLK1 antibody, a representative tuft cell marker, showed the number of tuft cells in the mice administered with succinate expanded compared to the control mice, consistent with the previous report (Fig. 6A) (Lei et al., 2018). Under this condition, the intracellular structures of the tuft cell were observed by TEM (Fig. 6B). Here, all 13 cells identified in the semi-thin sections were actually tuft cells with thick microvilli, and whole cell bodies were observable in 12 of these 13 cells. Furthermore, high-magnification observation showed the intracellular structures specific to tuft cells such as the microvilli (Fig. 6B,C), tubulovesicular system (Fig. 6C) and filamentous structures which extend from the tip of the cell to the periphery of the nucleus (Fig. 6B,C, white arrows). Golgi apparatus (Fig. 6D) and basal mitochondria (Fig. 6E) were also confirmed. To date, the existence of the secretory vesicles in the tuft cells to traffic IL25 has not been shown. We speculated that the basal vesicles are increased in the tuft cells by the stimulation with succinate, but no such characteristic changes were observed (Figs. 5E and 6F), which might mean that IL25 are released independently of the mechanism via secretory vesicles. To uncover the mechanism to secrete IL25, it would first be necessary to identify its intracellular localization by immuno-TEM. Furthermore, the length and thickness of the microvilli and the morphology of the tubulovesicular system were not changed (Figs. 5B,C and 6B,C,G,H). In summary, although no dramatic morphological changes in the tuft cell stimulated with succinate compared to steady state were observed, these results also suggest the technical utility of this new method for the observation of tuft cell in various conditions. To investigate the ultrastructural changes in the tuft cells between them in more detail, the 3D reconstruction by tomography would be required. Our novel CLEM protocol would be also useful in such analysis.

In electron micrograph, the tuft cell tends to be darker than adjacent cells (Fig. 5A). In order to investigate whether or not the dark tone is due to the Toluidine Blue staining and whether this disrupts the natural view of the tuft cell, ultrathin sections containing the tuft cells were prepared from the semi-thin sections without Toluidine Blue staining. For this analysis, the semi-thin sections of the mice administered with succinate were used, because the expansion of the tuft cell in this model increases the frequency of the tuft cells found on the ultrathin section without the need for the identification process by Toluidine Blue staining. The results showed that the dark tone in the tuft cells was also observable without Toluidine Blue staining (Fig. 6I and J). Furthermore, the structures including microvilli, the tubulovesicular system and filaments were compatible with the tuft cells stained with Toluidine Blue. Thus, these results indicate that the dark tone in the tuft cell is probably not due to the Toluidine Blue staining, and that the process does not disrupt the natural view of the tuft cell. It might be due to the dense structure of the tuft cells such as cytoskeletal components.

A possible limitation of this method is that the one or two sections are sometimes missing at the beginning of the next semi-thin section because the surface of the semi-thin block is sliced off before the ultra-thin sectioning. However, the loss of one or two ultrathin sections rarely misses the target structures of interest.

In conclusion, we showed that the CLEM strategy using serial semi-thin sections stained with Toluidine Blue can be applied in the case of low-frequency cells such as small intestinal tuft cells. Furthermore, we succeeded in re-embedding almost without any damage to the semi-thin sections by using Aclar film as opposed to glass slides. In particular, the semi-thin sections put on Aclar film are suitable for the preparation of the serial ultrathin sections, as shown in Fig. 4, due to their physical properties. It also means that this method can be principally used for the visualization of 3D structures in the tuft cell. In fact, the preparation of the serial ultrathin sections enables us to efficiently find various structures, especially small structures such as the centrosome and Golgi apparatus. The tuft-cell-specific structures such as thick microvilli, tubulovesicular system and distinguished filamentous structures were also confirmed. Although dramatic morphological changes in the tuft cell activated by succinate were not observed compared to that of steady state, this result suggests the versatility of this method for the observation of tuft cells in various conditions. Thus, this enhanced protocol would benefit future studies of tuft cells, their specific microstructures and physiological functions.

## MATERIALS AND METHODS

### Mice

C57BL/6J mice were maintained in specific pathogen-free conditions at the animal facilities of National Center for Global Health and Medicine, fed with CE-2 (CLEA Japan, Inc.) and kept under a 12:12 h light–dark cycle. All animal experimental procedures were approved by the Institutional Animal Care and Use Committee at the National Center for Global Health. For the experiment, adult mice (8–9 weeks, male) were used. For the stimulation of the tuft cell, 200 mM succinate (Nacalai Tesque, 32405-75) were administered to the mice for 7 days *ad libitum*.

### Immunohistochemistry

The small intestines were flushed with PBS and opened longitudinally. The tissues were fixed with 4% paraformaldehyde (Nacalai Tesque, 26126-25) in PBS for overnight at 4°C. For the frozen tissue sections, the tissues were then immersed in 20% (w/v) sucrose (Nacalai Tesque, 30403-55) overnight at 4°C, were embedded in OCT Compound (Sakura, 4583) and were sectioned at 5 μm on CryoStar™ NX70 Cryostat (Thermo Fisher Scientific). For the immunofluorescence of PFA fixed frozen sections, the sections were washed with PBS containing 0.1% TritonX-100 (FUJIFILM Wako Pure Chemical, 162-24755) (PBS-T) for 5 min twice. After the antigen retrieval using microwave treatment for 10 min in 10 mM citrate buffer (pH 6.0), the sections were blocked with Block Ace (DS Pharma Biomedical, UKB80) for 1 h at room temperature. The sections were then incubated with an anti-DCLK1 rabbit antibody (Abgent, AP7219b) diluted into 1:400 with 10-fold diluted Block Ace/PBS-T overnight at 4°C. After washing with 0.1% PBS-T for 5 min three times, the sections were incubated with Goat anti-Rabbit IgG (H+L) Cross-Adsorbed Secondary Antibody, Alexa Fluor 488 (Thermo Fisher Scientific, A11008) diluted into 1:500 with PBS-T containing Hoechst33342 for 1 h at room temperature. After washing with PBS-T for 5 min three times, the sections were mounted with 2.5% poly(vinyl alcohol) (Merck, P8136). The images were acquired using fluorescence microscopy (Leica, AF6000-DMI6B) (Objective lens; HCX PL FLUOTAR 20×/0.40 CORR PH1) (Camera; Leica DFC 350FX).

### TEM

After anesthesia with sevoflurane, the small intestine was harvested. The luminal contents were then removed by washing with PBS and the small intestine was opened longitudinally. After that, the tissue was cut into segments (∼1 cm) and fixed with 4% paraformaldehyde (Nacalai Tesque, 26126-25) and 0.2% glutaraldehyde (FUJIFILM Wako Pure Chemical, 072-02262) in PBS overnight at 4°C. After washing with PBS by the rotation for 10 min three times, the tissues were cut into small pieces (∼1 mm^3^) and the pieces was post-fixed with filtrated 2% OsO_4_ (TAAB, O001) in 30 mM HEPES buffer (pH 7.4) (Dojindo, GB70) containing 100 mM NaCl (FUJIFILM Wako Pure Chemical, 191-01665) and 2 mM CaCl_2_ (FUJIFILM Wako Pure Chemical, 038-24985) by the rotation for 1 h at 4°C. The osmium solution was filtrated using a syringe filter to remove the glasses powder which can be contaminated when the ampoule is opened because the contamination of the glass powder prevents the sectioning. After washing with water by the rotation for 10 min three times, the fixed tissues were dehydrated in a graded ethanol series (Muto Pure Chemical, 40262), infiltrated with propylene oxide (TAAB, P021), and embedded in Quetol 812 epoxy resin (Nisshin EM). Resin blocks were semi-thin sectioned at 1.5 μm thickness in ribbons with an ultramicrotome (Leica EM UC7, Leica). The ribbons of serially aligned sections were collected on an Aclar film (Nisshin EM, 453) and stained with 0.5% Toluidine Blue (Merck, T3260) in 0.5% sodium tetraborate buffer (FUJIFILM Wako Pure Chemical, 194-01415) for 10 s on the hotplate (80°C) to identify tuft cells (Fig. 3A). Tuft-cell-containing sections on the Aclar films were cut into each section (Fig. 3F) and re-embedded in NER-814 epoxy resin (Nisshin EM, 3951) individually. The Aclar films attached to the semi-thin sections are easily removed after the resin cured at room temperature (Fig. 3I). The re-embedded semi-thin sections were trimmed to a small area (∼200 μm×150 μm, Fig. 3J) and serially sectioned at 80 nm thickness with an ultramicrotome (Leica EM UC7, Leica, Fig. 3K). Thus, 15–20 ultrathin sections (80 nm) can be made with one semi-thin section (1.5 μm). Ribbons of ultrathin sections were picked up on Butvar B-98 (Nissin EM, ID02K) coated slot grids (Nissin EM, 2481) according to instructions in Handley and Olsen, 1979 (Fig. 4A), contrasted with 2% uranyl acetate (Merck, 8473) and lead citrate (TAAB, L018), and then observed with a transmission electron microscope (JEM-1400, JEOL Ltd., Tokyo, Japan).
